# A phase IB/IIA study of remestemcel‐L, an allogeneic bone marrow‐derived mesenchymal stem cell product, for the treatment of medically refractory ulcerative colitis: an interim analysis

**DOI:** 10.1111/codi.16239

**Published:** 2022-07-19

**Authors:** Amy L. Lightner, Neda Dadgar, Caroline Matyas, Kavita Elliott, Clifton Fulmer, Neha Khaitan, Justin Ream, Douglas Nachand, Scott R. Steele

**Affiliations:** ^1^ Department of Colorectal Surgery Digestive Disease and Surgery Institute, Cleveland Clinic Cleveland Ohio USA; ^2^ Department of Inflammation and Immunity Lerner Research Institute, Cleveland Clinic Cleveland Ohio USA; ^3^ Department of Pathology Robert J. Tomsich Pathology and Laboratory Medicine Institute, Cleveland Clinic Cleveland Ohio USA; ^4^ Department of Radiology Imaging Institute, Cleveland Clinic Cleveland Ohio USA

**Keywords:** Mayo score, mesenchymal stem cells, ulcerative colitis

## Abstract

**Aim:**

There have been no studies into the direct injection of mesenchymal stem cells (MSCs) for luminal ulcerative colitis (UC). Our aim was to investigate the efficacy of MSCs delivered locally via endoscopic delivery, as is done in the setting of perianal disease, to treat the local site of inflammation directly.

**Method:**

A phase IB/IIA randomized control clinical trial of remestemcel‐L, an *ex vivo* expanded allogeneic bone marrow‐derived MSC product, at a dose of 150 million MSCs versus placebo (2:1 fashion) delivered via direct injection using a 23‐gauge sclerotherapy needle at the time of colonoscopy was designed to assess the safety and efficacy of endoscopic delivery of MSCs for UC. The main outcome measures were adverse events, Mayo score and Mayo endoscopic severity score at 2 weeks, 6 weeks and 3 months post‐MSC delivery.

**Results:**

Six patients were enrolled and treated; four received MSCs and two placebo. All had been on prior anti‐tumour necrosis factor or anti‐integrin therapy. There were no adverse events related to MSCs. In the treatment group (*n* = 4), the Mayo endoscopic severity score decreased in all patients by 2 weeks after MSC delivery. At 3 months, all patients were extremely satisfied or satisfied with their MSC treatment based on the inflammatory bowel disease patient‐reported treatment impact (IBD‐PRTI), and treatment response was described as excellent or good in all patients. In the control group (*n* = 2), the Mayo endoscopic severity score did not increase as a result of being off alternative therapy. At 3 months, patients were dissatisfied according to the IBD‐PRTI, and treatment response was poor or unchanged.

**Conclusion:**

MSCs may offer a safe therapeutic option for the treatment of medically refractory UC. Early data suggest improved clinical and endoscopic scores by 2 weeks after MSC delivery.

## INTRODUCTION

Following the US Food and Drug Administration (FDA) approval of infliximab for the treatment of ulcerative colitis (UC) in 2005, monoclonal antibodies have become the cornerstone of medical therapy for the treatment of moderate to severe UC. While monoclonal antibodies have certainly changed the treatment paradigm for UC patients, they are fraught with potential side effects and their efficacy is limited by primary nonresponse and secondary loss of response [1, 2]. Even in the era of biologicals, up to 30% of UC patients still require major abdominopelvic surgery to remove the colon and rectum for medically refractory disease [3].

Mesenchymal stem cells (MSCs) were introduced as a safe and effective therapy for the treatment of perianal Crohn's disease (CD) in 2003 following an initial report of complete healing of a refractory rectovaginal fistula following local MSC delivery [4]. Several phase I [4], phase II [5, 6, 7] and phase III [8] clinical trials followed, underscoring the safety of local injection of MSCs for perianal CD. Secondarily, significant efficacy was described compared with placebo. A limited number of studies have assessed systemic delivery of MSCs for inflammatory bowel disease [9, 10, 11]. The only study of systemic MSCs was a phase I/II trial of 70 UC patients treated by delivery into a peripheral vein or into the superior mesenteric artery versus placebo. Clinical response and remission were increased in the treatment group compared with the control group, and there were no adverse reactions after MSC delivery [11]. Because it has been well established that there is significant pulmonary trapping following intravenous delivery of MSCs [12], we sought to investigate the efficacy of MSCs delivered locally via endoscopic delivery, as is done in the setting of perianal disease, to treat the local site of inflammation directly.

We designed a randomized phase IB/IIA clinical trial of the local delivery of remestemcel‐L, an *ex vivo* expanded allogeneic bone marrow‐derived MSC product, at the time of colonoscopy using a 23‐gauge sclerotherapy needle to inject cells directly into the submucosal layer of the colon and rectum. We herein describe the data for the first six patients at the primary endpoint of 3 months with regard to safety, clinical efficacy and endoscopic efficacy.

## METHOD

### FDA allowance to proceed

The FDA gave the allowance to proceed with a phase IB/IIA randomized clinical trial of medically refractory UC patients under IND#26555. An independent medical monitor was used throughout the course of the trial to assess patient safety and grade any adverse events.

### Institutional review board

Following FDA allowance to proceed, institutional review board (IRB) approval was granted by the Cleveland Clinic Foundation at Cleveland Clinic, OH under IRB #20–1005. The trial was then registered on clinical trials.gov under ClinicalTrials.gov identifier NCT04543994.

### Investigational agent

Remestemcel‐L is a product consisting of culture‐expanded mesenchymal stem cells (ceMSCs) isolated from the bone marrow of healthy adult donors. The final product is composed of MSCs formulated in Plasma‐Lyte A (70%), dimethyl sulfoxide (10%) and human serum albumin (HSA; 25%) solution (20%, comprising 5% HSA and 15% buffer) at a concentration of ≥6.68 × 10^6^ viable cells/ml. Each vial of product contains 3.8 ml of cryopreserved cell suspension (total cells per vial ≥25 × 10^6^). The product is stored at ≤−135°C in the vapour phase of liquid nitrogen until use. Final products must pass a panel of quality control release tests for safety, identity, purity and potency prior to use in humans. Additional information can be found in Chemistry, Manufacturing and Controls (CMC) by Mesoblast Inc. for manufacturing of remestemcel‐L (MF 16,678: CMC for allogeneic *ex vivo* ceMSCs). On the day of patient treatment, the bone marrow transplant laboratory at Cleveland Clinic thawed the prepared cells for injection once they received confirmation from the study team that the patient had arrived and evaluation of the patient in the preoperative area allowed for the procedure to proceed. A total of 6 (150 million dose) or 12 (300 million dose) vials were thawed in a 37°C water bath for 5 min. Upon thawing, the cells were transported by the cell therapy laboratory to the operating room where the sponsor investigator received the cells.

### Method of delivery

**FIGURE 1:**
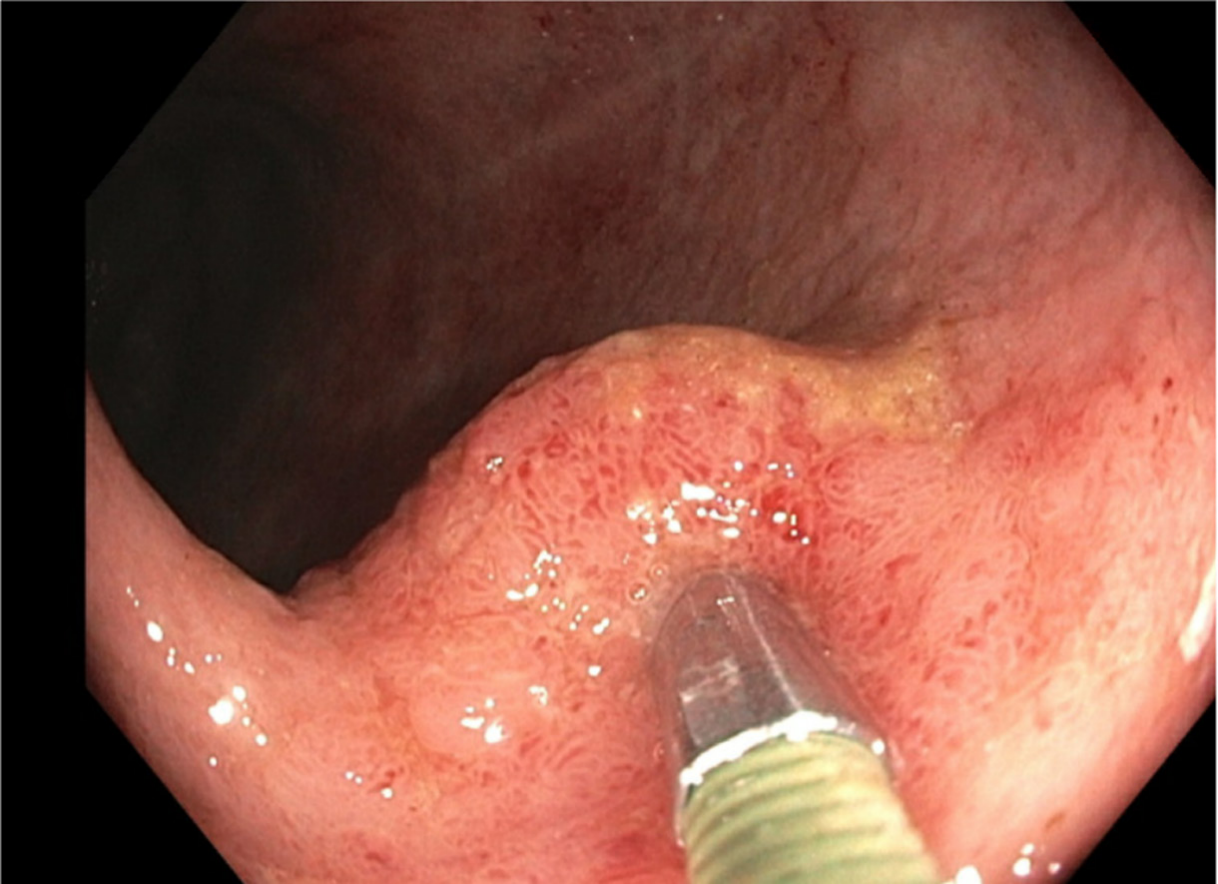
At the time of colonoscopy, a 23‐gauga sclerotherapy needle is used to inject the MSC product directly into the submucosal layer of the bowel wall.

Enrolled patients received either 150 million cells or control (saline) after randomization to treatment with MSC versus control (2:1) with Medidata Rave. Patients randomized to the treatment group were transported to the operating room, where they underwent a colonoscopy. During the procedure, an adult colonoscope was used through which a 23‐gauge single‐use sclerotherapy needle was used to inject the cells into the submucosal layer as evident by a small bleb raised in the mucosa. Delivery of MSCs was as follows: for the 150 million MSC dosage, 150 million cells were suspended in 22.8 ml, which were delivered into the submucosa of the caecum, proximal transverse colon, distal transverse colon descending colon, sigmoid colon and rectum, at 3.8 ml in each location. For placebo, 22.8 ml of saline was injected in the same locations as the 150 million MSC group (Figure 1).

### Study objectives

The primary objective was to determine the safety of endoscopic delivery of remestemcel‐L for the treatment of medically refractory UC. Safety was defined as a lack of adverse and serious adverse events related to the delivery procedure or investigational product.

The secondary objective was to assess luminal healing induced by one or two doses of remestemcel‐L at a dose of 150 million cells. Healing was defined by both clinical and endoscopic metrics. Clinical remission was defined as a Mayo Clinic score of 2 or lower and no subscore higher than 1, and mucosal healing was defined as an endoscopic subscore of 0 or 1. Endoscopic remission was defined as Mayo Clinic scale endoscopic subscore of 0 or 1. Clinical response was defined as reduction in the Mayo Clinic score by three points and a decrease of at least 30% from the baseline score with a decrease of at least two points on the rectal bleeding subscale to an absolute rectal bleeding score of 1 or 2. Endoscopic response was defined as a decrease in the Mayo Clinic endoscopic subscore by at least one point. Lack of response was defined as no improvement in the clinical or endoscopic scoring systems.

### Inclusion and exclusion criteria

A full list of inclusions and exclusions is given in the Supporting Information. Briefly, patients were included if they were adult (>18 years of age), had a diagnosis of UC for a duration of at least 6 months and had been nonresponsive or lost response to at least one monoclonal antibody. Exclusion criteria included presence of colonic neoplasia within 30 days of MSC therapy, pregnancy or lactation, having a diagnosis of CD, having a colonic stricture unable to be passed with a scope or having *Clostridium difficile* colitis or cytomegalovirus (CMV) colitis.

### Study design

This was an open label phase IB/IIA study of endoscopic delivery of remestemcel‐L for the treatment of medically refractory UC.

**FIGURE 2:**
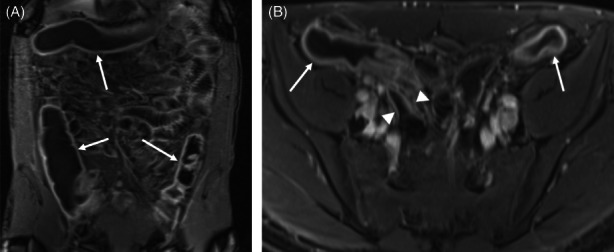
Magnetic resonance enterography (MRE): coronal (A) and axial (B) T1‐weighted fat‐saturated postcontrast images from a baseline MRE study in a 39‐year‐old patient with ulcerative pancolitis shows diffuse colitis with wall thickening and mural hyperenhancement (arrows) but normal appearance of the distal and terminal ileum (arrowheads).

Subjects were screened at outpatient clinic visits and interested qualified subjects were offered participation in the trial and consented. As part of standard of care for patients with UC, patients had a colonoscopy with biopsy and MR enterography to assess disease severity and the amount of involved colon if not done in the previous 30 days (Figure 2). The colonoscopy must have had an assigned Mayo Clinic endoscopic score and CMV and dysplasia ruled out; if not, colonoscopy was repeated.

If all clinical trial inclusion criteria were met, patients underwent a washout period of 4 weeks from their last dose of monoclonal antibody and 2 weeks from rectal corticosteroids or rectal 5‐aminosalicylic acid (5‐ASA) compounds. Patients on stable doses of 5‐ASA, corticosteroids (nonintravenous, oral at doses ≤20 mg), budesonide, immunomodulator therapy (azathioprine, methotrexate and 6‐mercaptopurine) for at least 4 weeks were allowed to continue at their stable dose. If recently discontinued, immunomodulators must have been stopped for at least 4 weeks, and budesonide, 5‐ASA compounds and oral corticosteroids for at least 2 weeks. Within the 7 days prior to MSC delivery, patients had a laboratory workup including a complete blood count with differential, sodium, potassium, chloride, bicarbonate, blood urea nitrogen, creatinine, albumin, pre‐albumin, C‐reactive protein (CRP), erythrocyte sedimentation rate (ESR) and *C. difficile* stool test. On the morning of the MSC injection procedure, patients underwent a general health assessment and abdominal examination and 24 h stool frequency, blood in stool and abdominal pain were assessed. MSCs were delivered at the time of colonoscopy on Day 0.

Patients then returned for follow‐up in the colon and rectal surgery clinic the next day (Day 1), 2 weeks after MSC delivery and 6 weeks after MSC delivery, where a physical examination was performed and serum laboratory values obtained. Flexible sigmoidoscopy with biopsies was also performed at the 2‐ and 6‐week visits. Three months following MSC delivery, if patients experienced clinical and endoscopic improvement they had the option of receiving a second dose of remestemcel‐L at a dose of 150 or 300 million MSCs (the same as the initial dose). Patients who had been in the placebo group crossed over at 3 months to receive MSC treatment at the time of colonoscopy (Tables 1 and 2).

**TABLE 1:** Study schedule for treatment group

	Visit 1	Visit 2 (Day 0)	Visit 3 (Day 1)	Visit 4 (Week 2)	Visit 5 (Week 6)	Visit 6 (Month 3)	Visit 7 (Month 6)	Visit 9 (Month 12)	Visit 11 (Month 24)
Visit window (days)	−30	0	0	±3	±3	±7	±14	±14	±104
Eligibility	X								
Informed consent	X								
Washout^a^	X								
Medical surgical history	X			X	X	X	X	X	X
Exam with vital signs	X	X	X	X	X	X	X	X	X
Mayo Clinic score	X	X		X	X	X	X	X	X
MRE	X^b^								
Colonoscopy with biopsy	X^b^	X^c^				X		X	X
Mayo Clinic endoscopic score	X	X		X	X	X	X	X	X
Flexible sigmoidoscopy				X	X		X		
Patient‐reported outcome surveys	X			X	X	X	X	X	X
MSC delivery		X				X^d^			
Pregnancy, urine^e^	X	X				X^f^			
AST/ALT	X								
Hepatitis panel	X								
HIV	X								
CBC	X			X	X	X	X	X	X
CMP	X			X	X	X	X	X	X
Pre‐albumin	X			X	X	X	X	X	X
CRP	X			X	X	X	X	X	X
ESR	X			X	X	X	X	X	X
*C. difficile*, faecal	X								
CMV colitis	X								
Calprotectin, faecal	X			X	X	X	X	X^g^	X^g^
Concomitant medications	X	X	X	X	X	X	X	X	X
Adverse events	X	X	X	X	X	X	X	X	X

*Abbreviations*: ALT, alanine aminotransferase; AST, aspartate aminotransferase; CBC, complete blood count; CMP, comprehensive metabolic panel; CMV, cytomegalovirus; CRP, C‐reactive protein; ESR, erythrocyte sedimentation rate; HIV, human immunodeficiency virus; MRE, magnetic resonance enterography; MSC, mesenchymal stem cell.

^a^ Washout period 2 weeks (5‐aminosalicylic acid, corticosteroids, immunomodulator therapy, azathioprine, methotrexate and 6‐mercaptopurine) or 4 weeks for biologicals (anti‐tumour necrosis factor, anti‐integrin and interleukin) will be initiated.

^b^ If not done clinically in the last 90 days.

^c^ Used to deliver MSCs or normal saline.

^d^ If not in clinical remission.

^e^ Obtained only for women of child‐bearing potential.

^f^ Obtained only if patient is going having a colectomy and is a woman of child‐bearing potential.

^g^ Labs only obtained if the patient did not have a colectomy performed.

**TABLE 2:** Study schedule for the control group

	Visit 1	Visit 2 (Day 0)	Visit 3 (Day 1)	Visit 4 (Week 2)	Visit 5 (Week 6)	Visit 6 (Month 3)	Visit 6.1 (Month 3; day 1)	Visit 6.2 (Month 3: Week 2)	Visit 6.3 (Month 3; week 6)	Visit 7 (Month 6)	Visit 8 (Month 9)	Visit 10 (Month 15)	Visit 11 (Month 24)
Visit window (days)	−30	0	0	±3	±3	±7	0	±2	±3	±7	±14	±14	±104
Eligibility	X												
Informed consent	X												
Washout^a^	X												
Medical surgical history	X			X	X	X		X	X	X	X	X	X
Exam with vital signs	X	X	X	X	X	X	X	X	X	X	X	X	X
Mayo Clinic score	X	X		X	X	X		X	X	X	X	X	X
MRE	X^b^												
Colonoscopy with biopsy	X^b^	X^c^				X^c^				X		X	X
Mayo Clinic endoscopic score	X			X	X	X		X	X	X	X	X	X
Flexible sigmoidoscopy				X	X			X	X		X		
Patient‐reported outcome surveys	X			X	X	X		X	X	X	X	X	X
Normal saline delivery		X											
MSC delivery						X				X^d^			
Pregnancy, urine^e^	X	X				X				X^f^			
AST/ALT	X												
Hepatitis panel	X												
HIV	X												
CBC	X			X	X	X		X	X	X	X	X	X
CMP	X			X	X	X		X	X	X	X	X	X
Pre‐albumin	X			X	X	X		X	X	X	X	X	X
CRP	X			X	X	X		X	X	X	X	X	X
ESR	X			X	X	X		X	X	X	X	X	X
*C. difficile*, faecal	X												
CMV colitis	X												
Calprotectin, faecal	X					X		X	X	X	X	X^g^	X^g^
Concomitant meds	X	X	X	X	X	X	X	X	X	X	X	X	X
Adverse events	X	X	X	X	X	X	X	X	X	X	X	X	X

*Abbreviations*: ALT, alanine aminotransferase; AST, aspartate aminotransferase; CBC, complete blood count; CMP, comprehensive metabolic panel; CMV, cytomegalovirus; CRP, C‐reactive protein; ESR, erythrocyte sedimentation rate; HIV, human immunodeficiency virus; MRE, magnetic resonance enterography; MSC, mesenchymal stem cell.

^a^ Washout period 2 weeks (5‐aminosalicylic acid, corticosteroids, immunomodulator therapy, azathioprine, methotrexate, and 6‐mercaptopurine) or 4 weeks for biologicals (anti‐tumour necrosis factor, anti‐integrin and interleukin) will be initiated.

^b^ If not done clinically in the last 90 days.

^c^ Used to deliver MSCs or normal saline.

^d^ If not in clinical remission.

^e^ Obtained only for women of child‐bearing potential.

^f^ Obtained only if the patient is going having a colectomy and is a woman of child‐bearing potential.

^g^ Labs only obtained if the patient did not have a colectomy performed.

### Histopathology and grading of inflammation

Haematoxylin and eosin‐stained tissue sections were prepared from formalin‐fixed, paraffin‐embedded tissue derived from endoscopically obtained rectal mucosal biopsies at baseline and at 2, 6 weeks and 3 months following MSC delivery or control. The degree of inflammation and mucosal injury was graded based on the simplified Geboes score for UC. Briefly, grade 0 was defined as no inflammatory activity, grade 1 as basal plasmacytosis, grade 2A as eosinophils in the lamina propria, grade 2B as neutrophils in the lamina propria, grade 3 as neutrophils in the epithelium and grade 4 as crypt and surface epithelial injury. The final score for each biopsy was determined by selecting the highest subgrade in the slide and tissue fragment showing the most severe disease activity. Scores greater than or equal to 3.1 (neutrophils in epithelium) were defined as active disease.

## RESULTS

### Patients enrolled

A total of six patients were enrolled and treated; four were randomized to treatment and two to placebo. All had been on previous anti‐tumour necrosis factor (TNF) or anti‐integrin therapy. Demographics are shown in Table 3.

**TABLE 3:** Demographic, medical and surgical history

	All (*n* = 6)	Control (*n* = 2)	Treatment (*n* = 4)
Sex: female	1 (16.7%)	1 (50.0%)	0 (0.00%)
Age (years)	37.2 (7.68)	34.5 (6.36)	38.5 (8.81)
Race			
Caucasian	5 (83.3%)	1 (50.0%)	4 (100%)
Indian	1 (16.7%)	1 (50.0%)	0 (0.00%)
Duration of disease (years)	14.0 [13.2;17.8]	13.0 [12.5;13.5]	16.5 [13.8;19.2]
Smoking	0 (0.00%)	0 (0.00%)	0 (0.00%)
Primary sclerosing cholangitis	0 (0.00%)	0 (0.00%)	0 (0.00%)
Small bowel disease	0 (0.00%)	0 (0.00%)	0 (0.00%)
Prior abdominal surgery	0 (0.00%)	0 (0.00%)	0 (0.00%)
Medications prior to study enrolment			
5‐ASA compounds	5 (83.3%)	2 (100%)	3 (75.0%)
Budesonide	2 (33.3%)	1 (50.0%)	1 (25.0%)
Corticosteroids/prednisone	3 (50.0%)	1 (50.0%)	2 (50.0%)
6‐MP, azathioprine/methotrexate	2 (33.3%)	1 (50.0%)	1 (25.0%)
Infliximab/adalimumab/certolizumab	5 (83.3%)	2 (100%)	3 (75.0%)
Vedolizumab	3 (50.0%)	1 (50.0%)	2 (50.0%)
Tofacitinib	1 (16.7%)	1 (50.0%)	0 (0.00%)
Most recent medications prior to enrolment			
5‐ASA compounds	1 (16.7%)	0 (0.00%)	1 (25.0%)
Budesonide	1 (16.7%)	0 (0.00%)	1 (25.0%)
Infliximab/adalimumab/certolizumab	3 (50.0%)	2 (100%)	1 (25.0%)
Vedolizumab	2 (33.3%)	0 (0.00%)	2 (50.0%)

*Abbreviations*: 5‐ASA, 5‐aminosalicylic acid; 6‐MP, mercaptopurine.

### Safety

There were no adverse or serious adverse events related to the product under investigation. During the first 3 months five patients experienced adverse events; three reported abdominal pain in the first 24 h that resolved without treatment, one reported knee pain and one reported perianal pain at 6 weeks due to a perianal fissure (Table 4).

**TABLE 4:** Adverse events

Subject	Adverse event	Time from MSC treatment	Treatment needed	Related to MSCs
1	Abdominal pain	Day 1	No	No
2	Abdominal pain	Day 1	No	No
3	Knee pain and sprain	Day 2	No	No
4	Abdominal pain	Day 1	No	No
5	Perianal pain from fissure	Week 6	No	No

*Abbreviation*: MSC, mesenchymal stem cell.

### Efficacy

**FIGURE 3:**
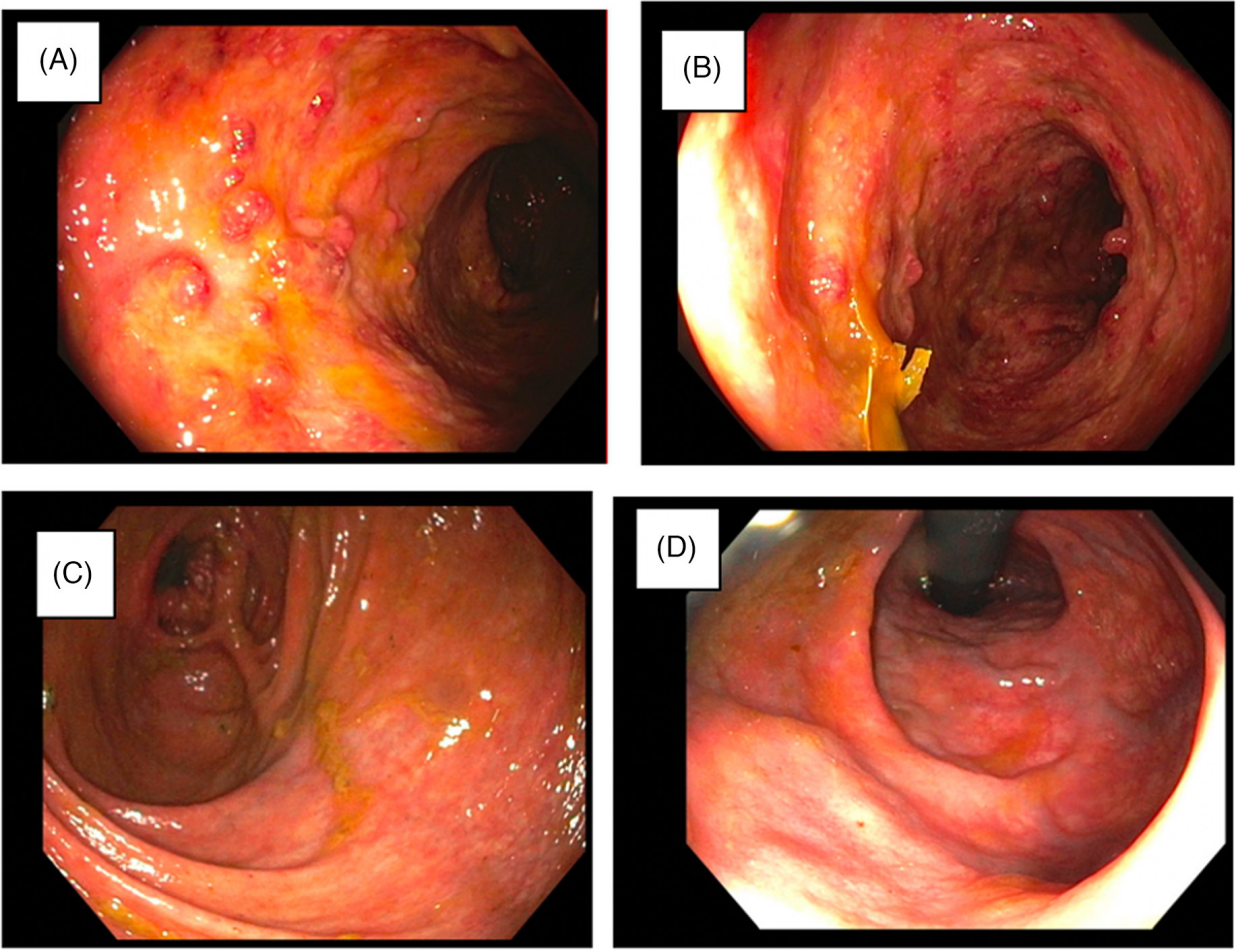
Colonoscopy: pretreatment colonoscopy with MSCs showing a Mayo score of 2 and pancolitis (A, B) as compared with the colonoscopy 3 months after MSC treatment showing a Mayo score of 0 to 1 throughout (C, D).

**FIGURE 4:**
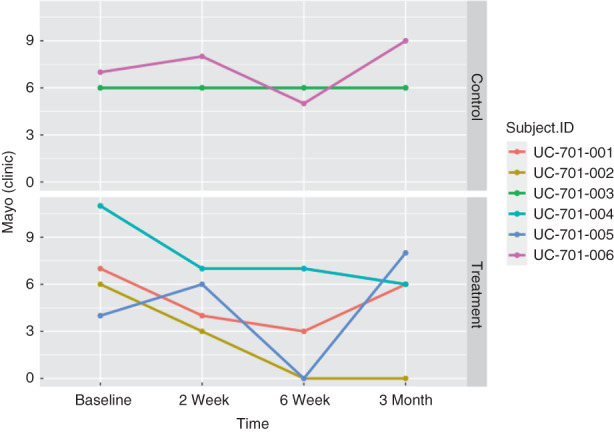
Mayo score in the treatment and control groups.

**FIGURE 5:**
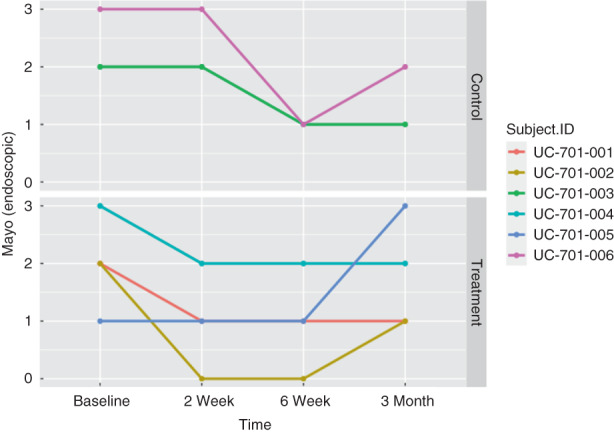
Mayo endoscopic severity score in the treatment and control groups.

In the treatment group (*n* = 4), the Mayo score and Mayo endoscopic severity score decreased in all patients by 2 weeks after MSC delivery (Figures 3, 4, 5). By 6 weeks after MSC treatment, all treated patients had achieved clinical and endoscopic remission as defined by the Mayo score and Mayo endoscopic severity score (Figures 3, 4, 5). CRP and faecal calprotectin remained relatively unchanged. The patient‐reported number of daily bowel movements decreased, as did urgency and presence of blood in the stool. With regard to patient‐reported outcomes, at 3 months, based on the IBD‐PRTI, all patients were extremely satisfied or satisfied with their MSC treatment. Treated response was described as excellent or good in all patients (Table 5).

**TABLE 5:** Treatment group outcomes

	Baseline (*n* = 4)	2 weeks (*n* = 4)	6 weeks (*n* = 4)	3 months (*n* = 4)
Mayo endoscopic severity score	2.00 [1.75;2.50]	1.00 [0.75;1.25]	1.00 [0.75;1.25]	1.50 [1.00;2.25]
Mayo score (clinical and endoscopic)	6.50 [5.50;8.00]	5.00 [3.75;6.25]	1.50 [0.00;4.00]	6.00 [4.50;6.50]
C‐reactive protein	0.50 [0.33;0.90]	1.10 [0.52;2.03]	0.65 [0.33;1.02]	1.10 [0.70;1.25]
Faecal calprotectin	244 [167;1407]	188 [28.1;350]	196 [115;209]	241 [211;885]
Bowel movements during day	6.50 [4.75;8.25]	4.50 [4.00;5.25]	4.00 [3.25;4.75]	3.50 [2.50;5.00]
Bowel movements at night	1.00 [0.00;2.25]	2.00 [1.50;3.00]	1.50 [1.00;2.50]	1.50 [0.75;2.50]
Urgency	4 (100%)	0 (0.00%)	1 (25.0%)	1 (25.0%)
Blood in stool	3 (75.0%)	2 (66.7%)	2 (50.0%)	2 (50.0%)
IBD‐PRTI				
1. extremely satisfied	–	2 (50.0%)	1 (25.0%)	1 (25.0%)
2. satisfied	–	0 (0.00%)	1 (25.0%)	3 (75.0%)
3. neither satisfied or dissatisfied	–	1 (25.0%)	1 (25.0%)	0 (0.00%)
4. dissatisfied	–	1 (25.0%)	1 (25.0%)	0 (0.00%)
Treatment response:				
Excellent	–	–	–	1 (25.0%)
Good	–	–	–	3 (75.0%)

*Abbreviation*: IBD‐PRTI, inflammatory bowel disease patient‐reported treatment impact.

In the control group (*n* = 2), the Mayo score did not decrease (Figure 5). CRP and faecal calprotectin remained relatively unchanged. The patient‐reported number of daily bowel movements increased, as did the number reporting blood in their stool; urgency remained unchanged. With regard to patient‐reported outcomes, at 3 months, based on the IBD‐PRTI, one patient was neutral and one patient was dissatisfied. Treated response was described as poor or unchanged in the control patients (Table 6).

**TABLE 6:** Control group treatment outcomes

	Baseline (*n* = 2)	2 weeks (*n* = 2)	6 weeks (*n* = 2)	3 months (*n* = 2)
Mayo endoscopic severity score	2.50 [2.25; 2.75]	2.50 [2.25; 2.75]	1.00 [1.00; 1.00]	1.50 [1.25; 1.75]
Mayo score (clinical and endoscopic)	6.50 [6.25; 6.75]	7.00 [6.50; 7.50]	5.50 [5.25; 5.75]	7.50 [6.75; 8.25]
C‐reactive protein	0.45 [0.38; 0.52]	1.10 [0.70; 1.50]	0.45 [0.43; 0.48]	0.75 [0.58; 0.92]
Faecal calprotectin	1514 [770; 2257]	128 [113; 143]	1514 [770; 2257]	–
Bowel movements during day	3.50 [2.75; 4.25]	5.00 [4.50; 5.50]	3.00 [2.50; 3.50]	6.00 [5.50; 6.50]
Bowel movements at night	0.50 [0.25; 0.75]	3.00 [1.50; 4.50]	0.00 [0.00; 0.00]	2.50 [1.25; 3.75]
Urgency	2 (100%)	1 (50.0%)	2 (100%)	2 (100%)
Blood in stool	1 (50.0%)	1 (50.0%)	1 (50.0%)	2 (100%)
IBD‐PRTI				
2. satisfied	–	0 (0.00%)	0 (0.00%)	0 (0.00%)
3. neither satisfied or dissatisfied	–	1 (50.0%)	1 (50.0%)	1 (50.0%)
4. dissatisfied	–	1 (50.0%)	1 (50.0%)	1 (50.0%)
Treatment response				
Fair	–	–	–	0 (0.00%)
Poor	–	–	–	1 (50.0%)
Unchanged	–	–	–	1 (50.0%)

*Abbreviation*: IBD‐PRTI, inflammatory bowel disease patient‐reported treatment impact.

### Histology

**FIGURE 6:**
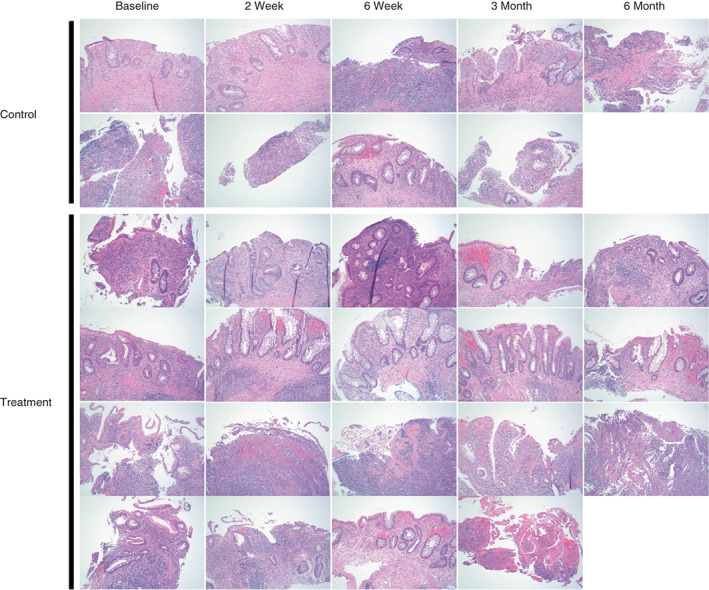
Histopathology: all patients showed evidence of rectal epithelial injury at baseline. Three months following the endoscopic delivery of MSCs, one of four patients showed mucosal healing with mildly increased lamina propria eosinophils and no evidence of active neutrophilic inflammation.

**FIGURE 7:**
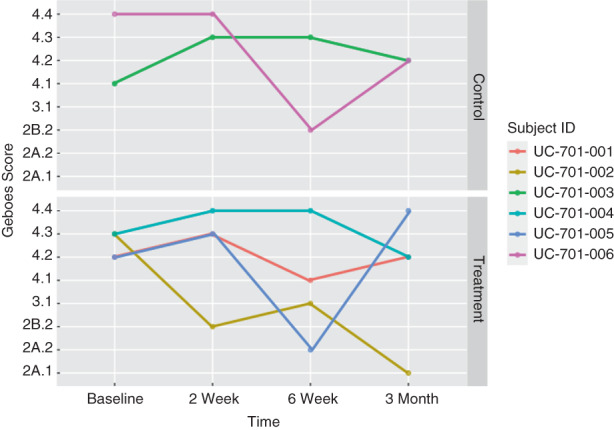
Geboes score in treatment and control groups.

At baseline, all patients showed histological evidence of epithelial injury (Figure 6). Rectal biopsies derived from one of the two patients in the control group showed attenuation of the surface and crypt epithelium (grade 4.1) while the other showed mucosal ulceration (grade 4.4). Similarly, baseline rectal biopsies from patients in the treatment group showed either probable or unequivocal crypt destruction and erosion (two patients grade 4.2, two patients grade 4.3). At 3 months after MSC treatment, one of the four patients (25%) showed no evidence of active disease, with only mildly increased lamina propria eosinophils, while the remaining three (75%) exhibited continued histological evidence of epithelial injury (Figure 7).

## DISCUSSION

Monoclonal antibodies have become the cornerstone of medical management for the treatment of moderate to severe UC. However, monoclonal antibodies have limited efficacy and significant risk of infective complications. Currently, with so many options, anti‐TNFα therapy (infliximab, adalimumab, certolizumab pegol), anti‐integrins (vedolizumab an anti‐α4β7 integrin), anti‐interleukins (ustekinumab, an anti‐interleukin‐12 and ‐23) and now small molecule inhibitors (tofacitinib), patients may cycle through numerous monoclonal antibodies when they lose response despite optimization. Unfortunately, each biological can take upward of 28 weeks to demonstrate clinical improvement in the maintenance phase [13]. Therefore, nonresponding patients may be left largely untreated during this period, becoming increasingly malnourished, anaemic and suffering from complications of their disease while awaiting evaluation of medical responsiveness. Due to these limitations, there has been a continued search for alternative treatment options that are safe and have equivalent efficacy to monoclonal antibodies. MSCs have gained attention as a safe and effective treatment option for perianal CD, a phenotype of inflammatory bowel disease that is notoriously difficult to treat [5, 6, 8, 14]. We therefore designed a phase IB/IIA trial to evaluate local targeted delivery of MSCs for the treatment of luminal UC.

Mesenchymal stem cells exist in almost all tissues [15, 16, 17], and are believed to reduce exacerbated inflammation due to their intrinsic immunomodulatory properties. Recently, the success of MSCs in treating severe inflammatory disorders, such as graft‐versus‐host disease [18], systemic lupus erythematosus [19], myocardial infarction [20], multiple sclerosis [21] and CD [22], has highlighted the therapeutic benefit of the immunomodulatory characteristics of MSCs [23, 24]. These immunomodulatory properties are thought to be mediated by migration to sites of active inflammation or tissue injury, secretion of anti‐inflammatory molecules such as interleukin‐10, hepatocyte growth factor, transforming growth factor β1 [25] and indoleamine 2,3‐dioxygenase [26] and paracrine signalling to nearby cells to maintain the local anti‐inflammatory environment [27, 28]. By influencing cytokine secretion profiles, MSCs can modulate the function of various immune cell types including lymphocytes, dendritic cells and macrophages. Therefore, the ability of MSCs to change the local inflammatory cell milieu, migrate to sites of inflammation and dampen immune responses underscores the escalating interest in using MSCs to treat inflammatory bowel disease.

Following two decades of early phase clinical trials using MSCs for perianal CD [5, 6, 7], darvadstrocel, an allogeneic adipose‐derived MSC product evaluated in two phase III randomized control trials [8, 29], has recently been approved by the regulatory bodies in Europe and Japan for the treatment of perianal CD. The mode of delivery of this product is direct injection in and around the fistula tract. Anecdotally, when we were treating patients in our own clinical trials of MSCs for the treatment of perianal fistulizing CD (ClinicalTrials.gov identifier NCT04519671) we noted that proctitis would also improve after direct injection near the internal opening of the fistula tract on the anal/rectal side. While previous clinical trials of the systemic delivery of MSCs for both luminal CD and UC have proven largely ineffective compared with conventional treatment approaches [9, 10, 11], none have used a method of direct targeted local delivery of cells, as has been utilized in clinical trials for perianal CD.

Remestemcel‐L has been used previously in a phase II clinical trial for luminal CD (NCT00294112) that randomized patients to receive two infusions 1 week apart of either 2 million cells or 8 million cells/kg intravenously. There were no reported infusion reactions, five mild adverse events unrelated to the investigational product and one serious adverse event (anaemia) felt to be unrelated to the investigational product. This phase II trial spurred a phase III clinical trial (Osiris protocol 610 NCT01233960) of four infusions over 2 weeks of either 600 million cells, 1200 million cells or placebo for medically refractory CD. The trial was stopped due to protocol design and a desire for open‐label therapy, but there were no safety concerns. There have been no studies, however, of remestemcel‐L for the treatment of UC and, furthermore, no studies of the direct local injection of MSCs for the treatment of luminal UC prior to this clinical trial. The use remestemcel‐L at a dose of 150 or 300 million cells given once or twice endoscopically had no adverse or serious adverse events related to the investigational product or the delivery of the investigational product using a 23‐gauge sclerotherapy needle at the time of colonoscopy, underscoring the safety of MSC therapy for UC.

With regard to efficacy, it has been well described that patients with prior exposure to anti‐TNF therapy have a lower response rate to subsequent monoclonal antibodies or small molecule inhibitors introduced compared to patients who are naïve to biologicals [30, 31, 32, 33]. This may be explained either by this group of patients being more treatment resistant in general or that chronic inflammation may leave the patient in a more ‘burnt out’ state. Thus, when trialling new therapeutics in a treatment‐refractory population, efficacy can be increasingly difficult to achieve with prior exposure to an increasing number of monoclonal antibodies and small molecule inhibitors. In our cohort of six patients, those who received treatment had a decrease in their Mayo endoscopic severity score and a decrease in symptoms, including daily bowel movements and urgency. This was seen by 2 weeks after MSC delivery. At 3 months after MSC delivery, a single patient also showed histological remission, with no neutrophilic inflammation or associated epithelial injury. Those in the control group did not show improvement in their symptoms or patient‐reported outcomes. In the treatment group, all patients chose to receive a second dose of MSCs at 3 months. Given that biologicals are dosed as an induction phase followed by a maintenance phase we will need to understand when patients lose response to MSC treatment to better determine how often we may need to re‐dose patients.

There are several limitations worth mentioning. First, and most importantly, this represents an interim analysis of a small number of patients. This limits our assessment of true efficacy in a larger cohort. Second, the protocol was not designed as a dose escalation study. There is still a paucity of data regarding optimal dosing and the potential need to re‐dose at more frequent dosing intervals, as is done with biological therapy. Third, the protocol utilized local delivery alone. Perhaps a combination of local and intravenous delivery could improve the overall efficacy of MSCs as a therapeutic option for UC. Lastly, the cohort treated represented a refractory cohort of patients who had been trialled on several biologicals in the past and had long‐standing disease. It is possible that treatment of earlier disease before the colon is ‘burnt out’ may be more effective for cell‐based therapeutics.

## CONCLUSION

MSCs offer a safe alternative therapeutic option for the treatment of medically refractory UC. Early data suggest improved clinical and endoscopic scores by as early as 2 weeks following MSC delivery.

## AUTHOR CONTRIBUTIONS

ALL: study design, patient recruitment, treatment of patients and manuscript writing. ND: data analysis and manuscript editing. CM: patient recruitment and data analysis. KE: patient recruitment and data analysis. CF: pathology reporting and data analysis. NK: pathology reporting and data analysis. JR: radiology reporting, data analysis and manuscript editing. DN: radiology reporting, data analysis and manuscript editing. SRS: study design, patient recruitment and manuscript editing.

## CONFLICT OF INTEREST

ALL is a consultant for Takeda, Mesoblast and Ossium.

## ETHICS STATEMENT

This study was approved by both Food and Drug Administration and the Instituation Review Board.

## Supporting information


Appendix S1

## Data Availability

All data can be made available by the first author upon email request.
