# Supplementary material for: A phase IB/IIA study of remestemcel‐L, an allogeneic bone marrow‐derived mesenchymal stem cell product, for the treatment of medically refractory ulcerative colitis: an interim analysis
Source: Colorectal Dis. 2022 Jul 19;24(11):1358–70. doi: 10.1111/codi.16239 (PMC9795998; doi:10.1111/codi.16239)
Supplement: Supplementary file 1 — Appendix S1 [file CODI-24-1358-s001.docx]

*Inclusion and Exclusion Criteria*

1. Males and Females 18-75 years of age.
2. Ulcerative colitis of at least 6 months duration with medically refractory symptoms
3. Exposure to corticosteroids, 5-ASA drugs, thiopurines, methotrexate, anti-TNF therapy, anti-integrin and anti-interleukin in the past are permitted but a washout period of 4 weeks for any monoclonal antibody is necessary.
   1. If receiving conventional immunomodulators (ie, AZA, 6-MP, or MTX), must have been taking them for ≥12 weeks, and on a stable dose for at least 4 weeks.
   2. If AZA, 6-MP, or MTX has been recently discontinued, it must have been stopped for at least 4 weeks.
   3. If receiving oral 5-ASA compounds, the dose must have been stable for at least 4 weeks.
   4. If receiving oral corticosteroids, the dose must be ≤20 mg/day prednisone or its equivalent and must have been stable for at least 4 weeks.
   5. If receiving budesonide, the dose must have been stable for at least 2 weeks.
   6. If oral 5-ASA compounds or oral corticosteroids (including budesonide) have been recently discontinued, they must have been stopped for at least 2 weeks.
4. The following medications/therapies must have been discontinued before first administration of study agent:
   1. TNF-antagonist therapy (eg, infliximab, etanercept, certolizumab, adalimumab, golimumab), vedolizumab, ustekinumab for at least 4 weeks.
   2. Cyclosporine, tacrolimus, or sirolimus, for at least 4 weeks.
   3. 6-thioguanine (6-TG) must have been discontinued for at least 4 weeks.
   4. Rectal corticosteroids (ie, corticosteroids [including budesonide] administered to the
   5. rectum or sigmoid colon via foam or enema or suppository) for at least 2 weeks.
   6. Rectal 5-ASA compounds (ie, 5-ASAs administered to the rectum or sigmoid colon via foam or enema or suppository) for at least 2 weeks.
   7. Parenteral corticosteroids for at least 2 weeks.
   8. Total parenteral nutrition (TPN) for at least 2 weeks.
   9. Antibiotics for the treatment of UC (eg, ciprofloxacin, metronidazole, or rifaximin) for at least 2 weeks.
5. No colonic dysplasia and malignancy as ruled out by colonoscopy within 30 days of MSC delivery
6. Ability to comply with protocol
7. Competent and able to provide written informed consent
8. Must have lost response to at least one monoclonal antibody (anti-TNF, anti-interleukin, or anti-integrin therapy), tofacitinib, or have a contra-indication to biologic therapy
9. If patient is of reproductive capacity, willing to use adequate birth control measures while they are in the study

Patients with the following characteristics were excluded

1. Inability to give informed consent.
2. Clinically significant medical conditions within the six months before administration of MSCs: e.g. myocardial infarction, active angina, congestive heart failure or other conditions that would, in the opinion of the investigators, compromise the safety of the patient.
3. Specific exclusions;
   1. HIV
   2. Hepatitis B or C
4. Abnormal AST or ALT at screening defined as AST >100 or ALT > 100
5. Abnormal basic laboratory values with the following cut-offs:
   1. Alkaline phosphate >200
   2. WBC >13
   3. Hemoglobin <7
   4. Platelets <50 or > 1 million
   5. Creatinine >1.5
6. History of cancer including melanoma (with the exception of localized skin cancers) within 5 years of study enrollment
7. Investigational drug within one year of study enrollment
8. Pregnant or breast feeding.
9. Fulminant colitis requiring emergency surgery
10. Concurrent active clostridium difficile infection of the colon
11. Concurrent CMV infection of the colon
12. Evidence of colonic perforation
13. Massive hemorrhage from the colon requiring emergent surgery
14. Crohn’s colitis or indeterminate colitis
15. Microscopic, ischemic or infectious colitis
16. Neoplasia of the colon and preoperative biopsy
17. Presence of an ostomy
18. Prior small bowel resection
19. Previous colonic resection
20. Colonic stricture that unable to pass an adult colonoscope
21. Active or latent tuberculosis
22. Unable to wean off corticosteroids
23. Patients with extra colonic ulcerative colitis including primary sclerosing cholangitis
24. Patients with history of or current evidence of alcohol or drug abuse or dependence, recreational use of illicit drug or prescription medications, or have use of medical marijuana within 90 days of study entry
25. Patients with known allergy to local anesthetics
26. Patients with a known allergy to DMSO, porcine and/or bovine proteins
27. Patients taking anticoagulant medications (e.g. warfarin, heparin) or clopidogrel (Plavix) to reduce the risk of bleeding/ hemarthrosis
28. If patient is of reproductive capacity, unwilling to use adequate birth control measures while they are in the study
